# Altered microstructure of the splenium of corpus callosum is associated with neurodevelopmental impairment in preterm infants with necrotizing enterocolitis

**DOI:** 10.1186/s13052-021-01197-z

**Published:** 2022-01-10

**Authors:** Jong Ho Cha, Jung-Sun Lim, Yong Hun Jang, Jae Kyoon Hwang, Jae Yoon Na, Jong-Min Lee, Hyun Ju Lee, Ja-Hye Ahn

**Affiliations:** 1 Department of Pediatrics, Hanyang University College of Medicine, 222-1 Wangsimni-ro Seongdong-gu, Seoul, 04763 South Korea; 2 Department of Family Medicine, Kangbuk Samsung Hospital, School of Medicine, Sungkyunkwan University, Seoul, South Korea; 3 Department of Biomedical Engineering, Hanyang University, Seoul, South Korea; 4 Clinical Research Institute of Developmental Medicine, Seoul Hanyang University Hospital, Seoul, South Korea

**Keywords:** Diffusion tensor imaging, Very-low-birth-weight infant, Necrotizing enterocolitis, Brain microstructure, White matter tracts, Corpus callosum, Neurodevelopmental impairment, The Bayley scales of infant and toddler development

## Abstract

**Background:**

Necrotizing enterocolitis (NEC) is a devastating disease in preterm infants with significant morbidities, including neurodevelopmental impairment (NDI). This study aimed to investigate whether NEC is associated with (1) brain volume expansion and white matter maturation using diffusion tensor imaging analysis and (2) NDI compared with preterm infants without NEC.

**Methods:**

We included 86 preterm infants (20 with NEC and 66 without NEC) with no evidence of brain abnormalities on trans-fontanelle ultrasonography and magnetic resonance imaging at term-equivalent age (TEA). Regional brain volume analysis and white matter tractography were performed to study brain microstructure alterations. NDI was assessed using the Bayley Scales of Infant and Toddler Development-III (BSID-III) at 18 months of corrected age (CA).

**Results:**

Preterm infants with NEC showed significantly high risk of motor impairment (odds ratio 58.26, 95% confidence interval 7.80–435.12, *p* < 0.001). We found significantly increased mean diffusivity (MD) in the splenium of corpus callosum (sCC) (*p* = 0.001) and the left corticospinal tract (*p* = 0.001) in preterm infants with NEC. The sCC with increased MD showed a negative association with the BSID-III language (*p* = 0.025) and motor scores (*p* = 0.002) at 18 months of CA, implying the relevance of sCC integrity with later NDI.

**Conclusion:**

The white matter microstructure differed between preterm infants with and without NEC. The prognostic value of network parameters of sCC at TEA may provide better information for the early detection of NDI in preterm infants.

## Background

Although standardized feeding protocols, including breast milk feeding, have been implemented, necrotizing enterocolitis (NEC) remains a devastating disease in the neonatal intensive care unit (NICU) that can lead to neurodevelopmental impairment (NDI) in preterm infants. The mechanism of NEC is multifactorial, including intestinal immaturity, hypoxic-ischemic injury, infection, and inflammation, which may impact brain development through immunological and neural pathways [1]. According to a previous study, the frequency of proven NEC (stage II or greater) varies, ranging from 3 to 7% [2]. Approximately one-third of the patients with NEC require surgical intervention and have a substantial mortality risk, ranging from 15 to 30% [2, 3]. Along with its impact on outcomes of NEC survivors, NDI in proven NEC has recently gained great interest [4–7].

Diffusion tensor imaging (DTI) provides useful information about microstructural changes in connectivity and myelination by detecting water molecule diffusion in tissues. There has also been a great deal of interest in using diffusion anisotropy as a marker for white matter tract integrity in DTI, reflecting structural and functional alterations in the developing preterm brain. We recently reported that delayed maturation of the middle cerebellar peduncles could predict NDI in preterm infants. However, infants with NEC were excluded, and their association with NDI should be evaluated [8]. Although outcomes related to prematurity illness have remarkably improved, NEC and its comorbidities are associated with severe NDI, especially in preterm infants with a birth weight of < 1500 g [6, 7]. However, little is known about the impact and clinical implications of NEC on brain development between birth and term-equivalent age (TEA) in terms of altered white matter maturation. There have been few attempts to elucidate whether NEC is associated with white matter maturation; however, few studies have shown confounding results, and their relationship has yet to be established [9, 10].

Herein, we hypothesized that NEC would be associated with NDI in preterm infants with delayed microstructural maturation. The objectives of the study were as follows: [1] to investigate whether NEC is associated with decreased brain volume and delayed microstructural maturation and [2] to compare the developmental outcomes between preterm infants with and without NEC at 18 months of corrected age (CA) and investigate its clinical relevance.

## Methods

### Study population

This was a prospective observational cohort study involving postnatal follow-up of preterm infants admitted to the Hanyang Inclusive Clinic for Developmental Disorders in Hanyang University, College of Medicine. Informed consent was obtained from parents of all the children included in this study. The inclusion criteria were as follows: [1] preterm birth weight < 1500 g and a diagnosis of NEC stage II or greater [2]; no evidence of congenital malformation [3]; no evidence of intrauterine growth retardation (IUGR) [4]; no evidence of any grade of intraventricular hemorrhage (IVH) and periventricular leukomalacia (PVL) [5]; no evidence of spontaneous intestinal perforation (SIP); and [6] availability of brain magnetic resonance imaging (MRI) at TEA. Demographic and clinical data were prospectively recorded, including maternal information, gestational age (GA), birth weight, delivery type, sex, histology of placenta, antenatal steroid, and Apgar score. Neonatal data including NEC, respiratory distress syndrome (RDS), culture-proven sepsis, hypotension, patent ductus arteriosus (PDA), bronchopulmonary dysplasia (BPD), retinopathy of prematurity (ROP), and total parenteral nutrition (TPN) duration were analyzed. BPD was diagnosed based on the need for oxygen support at 28 days of age and at 36 weeks of postmenstrual age (PMA) [11]. All trans-fontanelle ultrasound scans were evaluated for IVH and PVL by a single pediatric radiologist. Early trans-fontanelle ultrasound scans were performed within 3 days of birth and at 1 and 3 weeks after birth. At TEA between 36 and 41 weeks of PMA, we performed brain MRI and DTI to evaluate the structural brain network. Lastly, the Bayley Scales of Infant and Toddler Development-III (BSID-III) was performed at 18 months of CA with cognitive, language, and motor composite scores. The BSID-III test is composed of an average of 100 and a standard deviation of 15 in each composite score. The NEC group was defined as preterm infants who were diagnosed with NEC stage II or greater according to Bell’s Modified staging criteria [12]. The control group was defined as preterm infants without evidence of NEC, congenital malformations, IUGR, IVH, PVL, and SIP.

### MRI studies

MRI scans were performed for preterm infants at TEA during natural sleep using a 3.0-T MRI scanner (Philips real-time compact magnet 3.0-T MRI system; Achieva 3.0 T X-Series) with a 16-channel SENSE head coil. The T1-weighted images included sagittal and axial T1 spin-echo sequences (400/25/2, repetition time [TR]/echo time [ms]/signal intensity average) and axial T2 turbo spin-echo sequences (3000/100/1). Cushions were placed between the subject and the radiofrequency coil during image acquisition. DTI was performed using a single-shot spin-echo-planar sequence with a SENSE factor of 2 and an echo-planar imaging factor of 51 (TR/TE, 8100/75 ms; matrix size, 112 × 112; field of view, 224 mm; 74 axial sections). The slice orientation was axial with a 2.0-mm thickness and parallel to the anterior-posterior commissure line. Forty to fifty slices covered the entire hemisphere and brainstem. Fifteen directions using an electrostatic gradient model (b = 800) were used for diffusivity measurement. The subjects were well-fed before the scan, and sedative medications were not used.

### Brain volumes

**Fig. 1:**
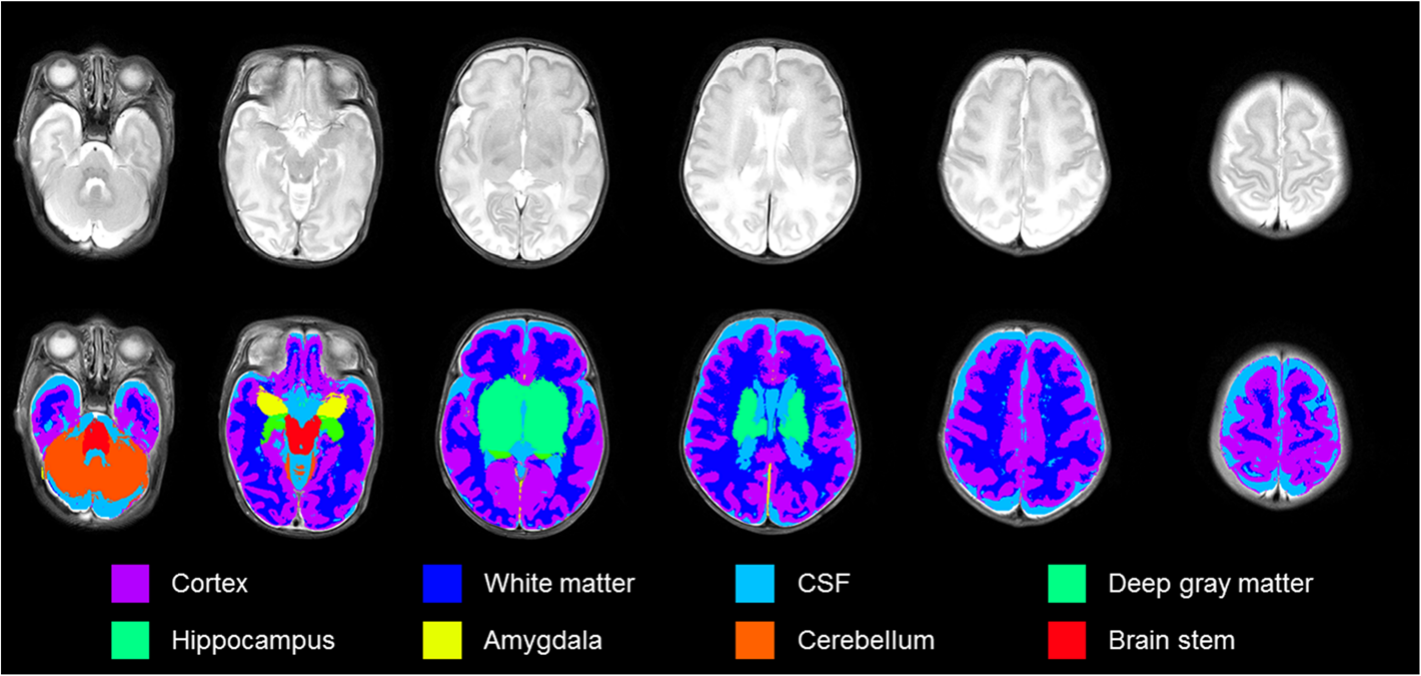
Example axial slices overlaid on T2-weighted images (1st row) and segmented into six brain morphological regions (2nd row). Subcortical gray matter includes hippocampus and amygdala. Abbreviations: CSF, cerebrospinal fluid

Brain volumes of preterm infants were measured using an advanced segmentation technique: Morphologically Adaptive Neonatal Tissue Segmentation (MANTiS; http://developmentalimagingmcri.github.io/man-tis), which is modified by Statistical Parametric Mapping (SPM) software [13]. It has modifications for neonatal imaging using neonate templates [14]. The pipeline classifies a T2-weighted MRI image of the brain into the following six sub-regions: cortical gray matter, cerebral white matter, cerebellum, subcortical gray matter (including deep nuclear gray matter, the hippocampus, and the amygdala), brainstem, and cerebrospinal fluid. Brain extraction was conducted using the Brain Extraction Tool (BET) in the FMRIB’s Software Library (FSL; http://www.fmrib.ox.ac.uk/). Then, using the “new segmentation tool” SPM 12, the initial tissue was classified with a neonate probability map included in MANTiS. Morphological watershed segmentation and filtering were processed against large ventricles or high-intensity white matter for reliable segmentation. Except for BET, the above processes were automatically executed using the MANTiS pipeline (Fig. 1).

### DTI processing

**Fig. 2:**
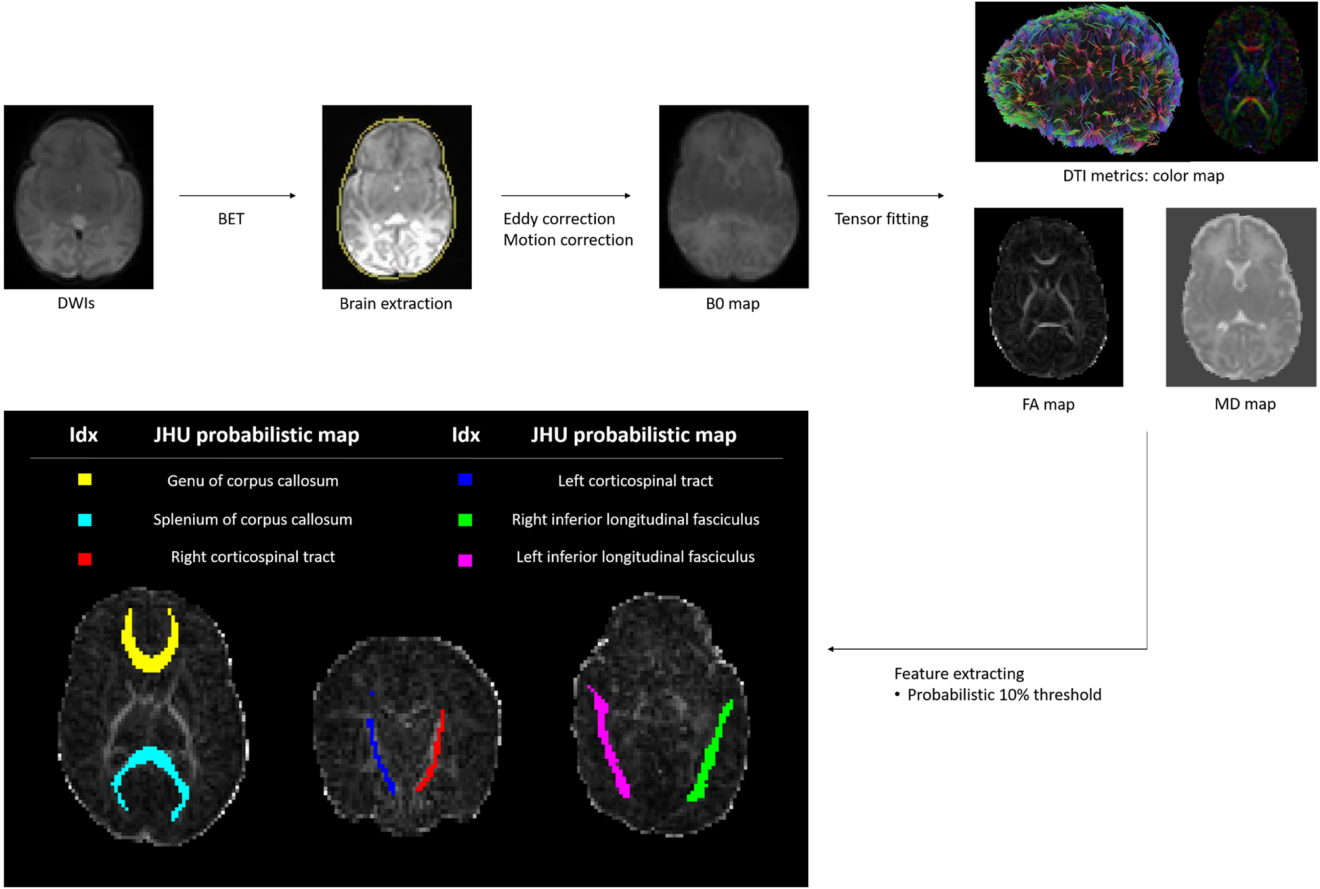
The pipeline of diffusion tensor image processing in this study. Abbreviations: DWI, diffusion weighted image; BET, brain extraction tool; DTI, diffusion tensor imaging; FA, fractional anisotropy; MD, mean diffusivity

**Fig. 3:**
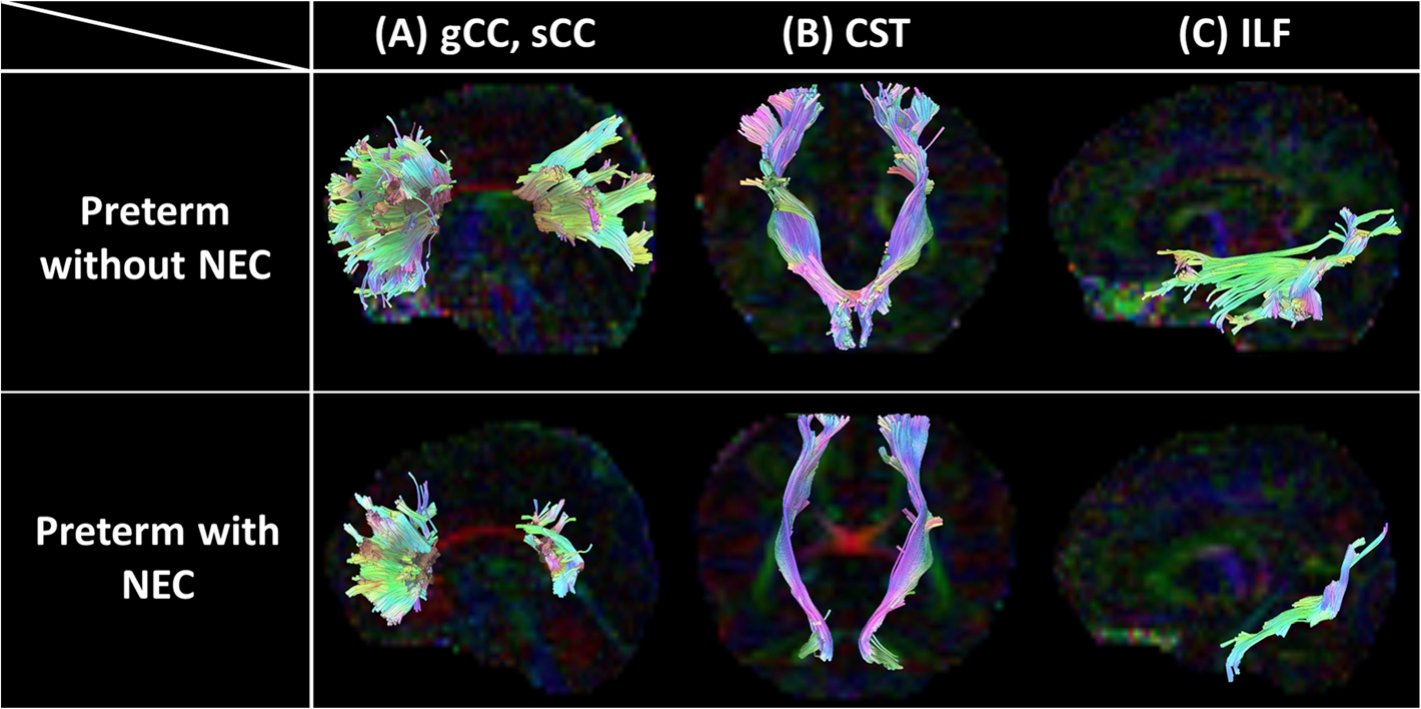
Tractographic reconstruction of segmented white matter. A single subject in each group exhibited the white matter integrity with FA threshold of 0.1. FA image registered on the JHU probabilistic maps (A) ~ (C) shows the gCC and sCC (A), CST (B), and ILF (C). Preterm with NEC showed shorter fibers toward occipital lobe than preterm without NEC at the sCC. In case of the CST, preterm with NEC exhibited smaller radiation range from cerebral peduncle to cerebral cortex (primary motor, premotor, somatosensory cortex). With respect to ILF, preterm with NEC had lesser fiber numbers toward the temporal lobe than the preterm without NEC. Compared to preterm infant without NEC, preterm with NEC (requiring surgery) had significant cognitive (58 points), language (45 points) and motor (40 points) impairment in 18 months of age. Abbreviations: FA, fractional anisotropy; NEC, necrotizing enterocolitis; gCC, genu of corpus callosum; sCC, splenium of corpus callosum; CST, corticospinal tract; ILF, inferior longitudinal fasciculus

The diffusion-weighted images were processed using the FMRIB’s Diffusion Toolbox (FDT) from FMRIB’s Software Library (Fig. 2). Motion artifacts and eddy current distortions were corrected by normalizing each diffusion-weighted image to a non-diffusion-weighted image (b0) using FMRIB’s linear image registration tool [15, 16]. BET was used to remove non-brain tissues [17]. Every voxel in the diffusion tensor was estimated using least-squares optimization [18]. A scalar map, including three values (λ1, λ2, λ3), fractional anisotropy (FA), and mean diffusivity (MD), was obtained. We used the probabilistic maps of the fiber pathways with a threshold of 0.1 based on the JHU-neonate atlas from Johns Hopkins University [19]. We used established probabilistic maps of fiber pathways that were constructed by DTI tractography and measured the average trace values of brain regions with a probability of more than 10%. The probabilistic map of white matter tracts was overlaid on the JHU-neonate atlas to quantify the FA and MD of the pathway-of-interest related to cognitive, language, and motor function. Regions of interest included the genu of the corpus callosum (gCC), splenium of the corpus callosum (sCC), left corticospinal tract (ltCST), right corticospinal tract (rtCST), left inferior longitudinal fasciculus (ltILF), and right inferior longitudinal fasciculus (rtILF). Furthermore, for a schematic understanding, we selected a single subject for each group and visualized the white matter integrity with threshold of 0.1 using a tractography software tool for MRI Analysis (DSI Studio, http://dsi-studio.labsolver.org/) (Fig. 3).

### Statistical analysis

Statistical analysis was performed using SPSS (version 21.0; SPSS, Chicago, IL, USA). Preterm infants were sub-categorized into two groups: preterm infants with NEC and without NEC. The demographic and clinical characteristics were compared using the Student’s t-test and Mann-Whitney U test continuous variables and Fisher’s exact test for categorical variables. We used a general linear model to compare differences in brain volumes, controlling for sex, GA, PMA at MRI, and intracranial volume after correction for multiple comparisons. Differences in network parameters between groups were analyzed using a general linear model after adjusting for sex, GA, and PMA at MRI. Comparing differences in network parameters, we conducted an additional two-step analysis, controlling for GA and PMA at MRI for the first step and included TPN duration for the second step for controlling nutritional aspect as covariates using Quade’s nonparametric rank analysis of covariance. In brain volume and network parameter analysis, Bonferroni correction was performed to reduce errors due to multiple comparisons. In developmental assessment, multivariate logistic regression model was used to investigate significant risk factors of NDI. Preterm infants with less than 70 (< − 2 standard deviation) in each composite score (cognition, language, motor) were defined as NDI. In addition, of network parameters that were statistically significant in the NEC group, we conducted multiple linear regression analysis of the association between network parameters and a composite score of BSID-III, including GA and PMA at MRI as covariates. Variables with *p* value < 0.05 were considered significant.

## Results

### Clinical characteristics

Table 1 presents the baseline demographics and clinical characteristics of the study population. Of the 109 preterm infants, we excluded 23 preterm infants and the detailed features were as follows: eight infants were diagnosed with IVH and PVL during NICU admission; six infants were lost to follow-up, nine infants had an insufficient quality of MRI imaging. In total, 86 preterm infants (20 infants with NEC and 66 infants without NEC) were included. Of the 20 infants diagnosed with NEC, three were diagnosed with severe NEC and required surgery. The mean GA of the NEC group and without NEC group was 27.03 and 28.88 weeks, respectively (*p* = 0.026). The mean PMA at MRI was 37.90 weeks in the NEC group and 36.91 weeks in the without NEC group (*p* = 0.210). Regarding neonatal characteristics, the NEC group had a significantly longer TPN duration (*p* = 0.003). Lastly, other neonatal morbidities, including RDS, BPD, PDA, and ROP, did not show significant differences.

**Table 1:** Baseline demographic characteristics of preterm infants

Variables	Without NEC (*n* = 66)	With NEC (*n* = 20)	*p* value
**Maternal characteristics**			
Maternal age (years)	34.14 ± 4.36	33.90 ± 5.12	0.857^*^
Mother’s education			0.134
High, n (%)	3 (4.5)	2 (10)	
Intermediate, n (%)	52 (78.8)	16 (80)	
Low, n (%)	11 (16.7)	2 (10)	
GDM (%)	7 (10.6)	0 (0)	0.193
PIH (%)	9 (13.6)	3 (15)	0.100
PPROM (%)	34 (51.5)	6 (30)	0.126
Histologic chorioamnionitis (%)	33 (50.0)	11 (55.0)	0.909
**Neonatal characteristics**			
Gestational age (weeks)	28.88 ± 2.65	27.03 ± 3.04	0.026^*^
Birth weight (g)	1202.20 ± 256.40	985.50 ± 305.62	0.008
Cesarean section (%)	52 (78.8)	18 (90)	0.340
Male sex (%)	35 (53)	12 (60)	0.618
Apgar score at 1 min	3.52 ± 1.69	2.70 ± 1.42	0.034^*^
Apgar score at 5 min	6.42 ± 1.19	5.35 ± 1.42	0.001^*^
Antenatal corticosteroid (%)	47 (71.2)	16 (80.0)	0.569
Postnatal corticosteroid (%)	14 (21.2)	4 (20.0)	1.000
RDS (%)	57 (86.4)	20 (100)	0.109
Culture proven sepsis (%)	31 (47.0)	14 (70)	0.126
Hypotension (%)	4 (6.1)	2 (10)	0.620
Severe NEC (Bell stage ≧ 3) (%)	0	3 (15)	0.011
PDA (%)	39 (59.1)	16 (80)	0.114
BPD moderate and severe (%)	14 (21.2)	5 (25)	0.762
ROP (%)	30 (45.5)	10 (50)	0.377
TPN days	32.98 ± 26.94	52.00 ± 28.58	0.003^*^
**Infants characteristics on PMA at MRI**			
PMA at MRI (weeks)	36.91 ± 1.71	37.90 ± 2.45	0.210^*^
Weight at MRI (g)	2515.91 ± 514.12	2517.00 ± 730.57	0.368^*^
Height at MRI (cm)	45.97 ± 3.41 (*n* = 65)	45.93 ± 4.04	0.697^*^
Head circumference at MRI (cm)	33.18 ± 1.82	32.88 ± 1.67	0.380^*^

Data are expressed as mean ± standard deviation or number (%)

Abbreviations: *NEC* necrotizing enterocolitis, *GDM* gestational diabetes mellitus, *PIH* pregnancy-induced hypertension, *PPROM* preterm premature rupture of the membranes, *PDA* patent ductus arteriosus, *BPD* bronchopulmonary dysplasia, *ROP* retinopathy of prematurity, *TPN* total parenteral nutrition, *PMA* postmenstrual age, *MRI* magnetic resonance imaging, *RDS* respiratory distress syndrome

^*^For non-normal distribution, Mann-Whitney U Test was used to compare the groups

### Brain volume

Table 2 shows the comparisons of brain volume analysis between the two groups. In every sub-region, preterm infants with NEC showed volume reduction compared to the control group. However, its reduction was not statistically significant after Bonferroni correction. Moreover, its insignificance remained after adjusting for several covariates including sex, PMA at MRI and total intracranial volume.

**Table 2:** Regional and total brain volume of preterm infants with and without NEC

Region of interest	Without NEC (*n* = 66)	With NEC (*n* = 20)	*p* value	*Adjusted p* value^*^
Cortical gray matter, cm^3^	147.61 ± 41.97	140.80 ± 34.83	0.545	0.116
Cerebral white matter, cm^3^	124.58 ± 67.00	102.72 ± 11.62	0.022	0.512
Deep gray matter, cm^3^	23.59 ± 5.21	21.30 ± 3.25	0.034	0.078
Hippocampus, cm^3^	2.65 ± 1.22	2.23 ± 0.72	0.180	0.146
Amygdala, cm^3^	1.34 ± 0.69	1.07 ± 1.03	0.214	0.533
Cerebellum, cm^3^	20.77 ± 9.80	18.84 ± 5.19	0.439	0.448
Brain stem, cm^3^	5.76 ± 1.63	5.05 ± 1.22	0.104	0.182
Cerebrospinal fluid, cm^3^	78.53 ± 32.50	61.99 ± 15.68	0.047	0.144
Total intracranial volume, cm^3^	404.83 ± 109.82	354.00 ± 40.54	0.027	0.102^‡^

Data are expressed as mean ± standard deviation

^*^General linear model with the group as a fixed factor, sex, PMA at MRI, and total intracranial volume as covariates

^‡^Adjusted by Gestational age, head circumference, and PMA at MRI

The significance level was corrected from 0.05 to 0.005 (0.05/9) by the Bonferroni correction method

Abbreviations: *NEC* necrotizing enterocolitis, *PMA* postmenstrual age, *MRI* magnetic resonance imaging

### DTI analysis

Representative images of the DTI analysis in this study are shown in Fig. 1. Table 3 shows the network parameters between the two groups. The MD of sCC was significantly higher (*p* = 0.001) in the NEC group after controlling for covariates. CST resulted in hemispheric differences, showing that MD of ltCST was significantly higher than that in the NEC group (*p* = 0.001). Lastly, in ILF, network parameters in both hemispheres were not different between preterm infants with and without NEC.

**Table 3:** Network parameters of diffusion tensor analysis in preterm infants with and without NEC

Region of interest	Without NEC (*n* = 66)	With NEC (*n* = 20)	Unadjusted *p* value^*^	Adjusted *p* value^**^	Adjusted *p* value + TPN days^‡^
gCC					
FA	0.181 ± 0.027	0.175 ± 0.012	0.841	0.509	0.386
MD	1.434 ± 0.129	1.445 ± 0.107	0.883	0.399	0.280
sCC					
FA	0.185 ± 0.032	0.173 ± 0.013	0.109	0.098	0.129
MD	1.446 ± 0.151	1.543 ± 0.108	0.002	0.001	0.001
ltCST					
FA	0.261 ± 0.041	0.257 ± 0.032	0.891	0.207	0.221
MD	1.230 ± 0.110	1.297 ± 0.082	0.003	0.001	0.001
rtCST					
FA	0.267 ± 0.038	0.261 ± 0.031	0.473	0.070	0.068
MD	1.296 ± 0.107	1.303 ± 0.122	0.720	0.129	0.160
ltILF					
FA	0.181 ± 0.028	0.170 ± 0.015	0.109	0.047	0.038
MD	1.484 ± 0.139	1.487 ± 0.101	0.620	0.029	0.743
rtILF					
FA	0.182 ± 0.027	0.174 ± 0.014	0.306	0.107	0.122
MD	1.406 ± 0.132	1.407 ± 0.117	0.736	0.586	0.515

Data are expressed as mean ± standard deviation

^*^For non-normal distribution, Mann-Whitney U Test was used to compare the groups

^**^Adjusted for gestational age and PMA at MRI; Quade’s nonparametric rank analysis of covariance was used to compare the groups

^‡^Adjusted by gestational age, PMA at MRI, and TPN days; Quade’s nonparametric rank analysis of covariance was used to compare the groups

The significance level was corrected from 0.05 to 0.008 (0.05/6) by the Bonferroni correction method

Abbreviations: *NEC* necrotizing enterocolitis, *FA* fractional anisotropy, *MD* mean diffusivity, *gCC* corpus callosum genu, *sCC* corpus callosum splenium, *ltCST* left corticospinal tract, *rtCST* right corticospinal tract, *ltILF* left inferior longitudinal fiber, *rtILF* right inferior longitudinal fiber, *PMA* postmenstrual age, *MRI* magnetic resonance imaging, *TPN* total parenteral nutrition

### Developmental outcomes

Table 4 presents the neurodevelopmental outcome of preterm infants with and without NEC. Of the 86 preterm infants with MRI analysis, 75 (87%) infants underwent BSID-III in 18 months of CA (19 with NEC and 56 without NEC). Our logistic regression model showed that NEC was a significant risk factor in motor impairment (odds ratio 58.26, 95% confidence internal 7.80–435.12). Looking into the BSID composite scores, the preterm with NEC group had significantly lower BSID-III scores in cognitive (87.00 ± 15.52 vs. 99.04 ± 13.95, *p* = 0.002), language (82.03 ± 14.03 vs. 92.45 ± 16.42, *p* = 0.016), and motor (74.42 ± 15.75 vs. 100.36 ± 14.41, *p* < 0.001) function than those of control group. We performed multivariate linear regression analysis between each composite score of BSID-III and MD of the tract, which was significant in tractography analysis. MD of sCC was significantly negatively associated with language score (*p* = 0.025) and motor score (*p* = 0.002). Likewise, in the ltCST, MD was marginally related to motor score (*p* = 0.058) (Table 5).

**Table 4:** Developmental assessment of preterm infants at 18 months of corrected age*

Variables	Cognitive impairment		Language impairment		Motor impairment	
	Odds ratio (95% C.I)	*p* value	Odds ratio (95% C.I)	*p* value	Odds ratio (95% C.I)	*p* value
NEC	1.95 (0.20–18.79)	0.561	1.50 (0.29–7.59)	0.622	58.26 (7.80–435.12)	< 0.001
GA (weeks)	0.92 (0.59–1.43)	0.726	0.85 (0.62–1.17)	0.321	1.20 (0.85–1.70)	0.290
PMA at MRI (weeks)	1.11 (0.69–1.85)	0.649	1.23 (0.87–1.76)	0.238	0.98 (0.65–1.47)	0.932
Sex (Male)	8.25 (0.57–57.30)	0.137	1.78 (0.45–7.04)	0.411	2.57 (0.54–12.25)	0.236
BPD moderate and severe	1.78 (0.12–25.60)	0.184	10.43 (0.75–144.1)	0.080	0.23 (0.01–2.92)	0.262
Maternal education**	0.49 (0.04–5.81)	0.572	0.53 (0.08–3.21)	0.491	2.87 (0.26–31.21)	0.387

Abbreviations: C. I, confidence interval; NEC, necrotizing enterocolitis; GA, gestational age; PMA, postmenstrual age; MRI, magnetic resonance imaging; BPD, bronchopulmonary dysplasia

^*^Includes 75 preterm infants (19 with NEC, 56 without NEC) out of 86 preterm infants

**Defined as mothers with college graduate or higher educational level

**Table 5:** Multiple regression analysis for the BSID-III composite scores in preterm infants*

BSID-III composite scores	MD, sCC (10^**−3**^ mm^**2**^/s)		GA		PMA at MRI	
	Coefficient (SE)	*p* value	Coefficient (SE)	*p* value	Coefficient (SE)	*p* value
Cognitive score	−18.414 (10.791)	0.092	1.209 (0.580)	0.041	0.665 (0.842)	0.432
Language score	−28.336 (12.402)	0.025	0.405 (0.667)	0.546	−0.131 (0.967)	0.892
Motor score	−41.913 (13.254)	0.002	1.080 (0.713)	0.134	−0.425 (1.034)	0.682
	**MD, ltCST (10**^**−3**^ **mm**^**2**^**/s)**		**GA**		**PMA at MRI**	
	Coefficient (SE)	*p* value	Coefficient (SE)	*p* value	Coefficient (SE)	*p* value
Cognitive score	−7.840 (15.431)	0.613	1.276 (0.591)	0.034	0.696 (0.870)	0.426
Language score	−22.770 (17.840)	0.206	0.469 (0.683)	0.495	−0.198 (1.006)	0.845
Motor score	−37.421 (19.380)	0.058	1.161 (0.742)	0.122	−0.564 (1.093)	0.608

All regression analyses for each Bayley composite score included MD (sCC or ltCST), GA, and PMA at MRI scan

Abbreviations: *BSID-III* Bayley Scales of Infant and Toddler Development-III, *MD* mean diffusivity, *sCC* corpus callosum splenium, *GA* gestational age, *PMA* postmenstrual age, *SE* standard error, *ltCST* left corticospinal tract, *MRI* magnetic resonance imaging

^*^Includes 75 preterm infants (19 with NEC, 56 without NEC) out of 86 preterm infants

## Discussion

To the best of our knowledge, this is the first study to address the association between delayed white matter maturation and NDI in preterm infants with NEC and without apparent brain abnormalities. Compared with preterm infants without NEC, the NEC group had a significant NDI at 18 months of CA. DTI analysis showed that the NEC group had increased MD of sCC and ltCST, indicating delayed microstructural maturation at TEA. Moreover, MD of sCC was negatively associated with BSID-III language and motor composite scores.

We found that the NEC group had smaller white and deep gray matter size and decreased total brain volume although the associations were not statistically significant. We assume that impaired brain volume in preterm infants with NEC in previous studies is due to confounders, including total intracranial volume or head circumference [20, 21]. Although the associations between NEC and brain volume were not evident, the results implied that brain volume reduction in the NEC group was due to delayed brain growth rather than secondary atrophy or brain injury caused by NEC.

In developmental assessment, NEC was a significant risk factor of NDI only in motor function. Considering our study set cut-off value of NDI with composite score under − 2 standard deviation, our result might have reflected moderate to severe NDI. As NEC group had significantly poor BSID composite scores in every index, further study with large sample size would be needed to elucidate its relationship. This study showed that preterm infants with NEC exhibited white matter maturation delay as network parameter distinctions in DTI analysis. Maturation delay of sCC and ltCST with prolonged MD was prominent, reflecting that neuronal fibers were loosely connected and thereby poorly integrated in the NEC group. This implies that NEC plays a substantial role in white matter maturation, along with prematurity, contributing to NDI. The corpus callosum (CC) is the largest white matter bundle that conducts inter-hemispheric information. In particular, sCC is the most integrated lesion connecting the temporal, parietal, and occipital cortices. It plays a major role in the transhemispheric processing of visual and acoustic data and is myelinated at 3–4 months of age [22, 23]. In previous DTI studies, delayed maturation of CC was implicated in cognitive function [22, 24], gait, and motor coordination [24] of preterm infants. Our study is in line with those studies, emphasizing that maturation of CC is associated with NDI in preterm infants with NEC. Moreover, we found that MD of sCC could be a biomarker of later NDI, especially in cognitive and language functions, reflecting its correlation with BSID-III composite scores.

Interestingly, an increase in MD was not followed by a decrease in the FA in the CC. Although both FA and MD are primarily calculated parameters that reflect the degree of myelination, they can contain different characteristics. FA measures the degree of anisotropy within a voxel, implying the direction of myelination, whereas MD measures the average degree of water molecule diffusion [25]. CC is myelinated at 3–4 months of age, suggesting less anisotropy and less myelination at TEA in this study. Thus, we assumed that delayed maturation of the NEC group would manifest with an increase in MD as early as TEA rather than a decrease in FA [26]. Given that altered brain development underpins maturation-dependent vulnerability, the insignificant difference between the two groups in the myelination of gCC could be explained by the general myelination pattern in the posterior-to-anterior direction [25].

The observed association between white matter maturation and NEC may reflect multifactorial etiologic factors of NEC, including intestinal immaturity, hypoxic-ischemic injury, infection, and inflammation. In particular, inflammation has been hypothesized to be the principal cause of NDI. Previous studies have shown that inflammatory conditions accompanied by sepsis are associated with altered brain microstructural perturbing white matter microstructural integrity [27, 28]. Moreover, Alshaikh et al. [29] showed that inflammatory conditions played a crucial role in white matter abnormalities, even in the absence of evident brain injury in a meta-analysis. Let alone the abnormalities in the infant period, Dubner et al. [30] showed that inflammatory conditions in the neonatal period had altered white matter microstructure in CC with delayed cognitive function at 6 years of age. As NEC has been implicated in the pathogenesis of infection and inflammation in one of the neonatal diseases, our results are in line with previous reports emphasizing the role of inflammation in NDI of infants with NEC. The mechanism of white matter delayed maturation in NEC is not well understood. However, multiple studies have reported elevated levels of inflammatory cytokines and disruption of the blood-brain barrier in animal models of NEC [31, 32]. We assume that the systemic inflammatory response causes widespread oxidative stress in the brain as NEC progresses. Subsequently, pre-oligodendrocytes are injured, which has marked vulnerability to cytokine injury and reactive oxygen, in response to the activation of astrocytes and microglia. Injured pre-oligodendrocytes may have failed to evolve into myelin-producing oligodendrocytes, thereby causing delayed white matter maturation and NDI [33]. Moreover, conditions followed by NEC including acidemia, sepsis and management followed by NEC including mechanical ventilation, systemic antibiotics and indwelling catheters may have worsened systemic inflammation and disrupted gut microbiota for early brain development.

Growing evidence suggests that early postnatal growth and nutrition during the NICU period affect brain volume expansion and white matter maturation [4, 34, 35]. These findings suggest that nutrition and growth are possible confounding factors of NDI in infants with NEC. To reflect their confounding effects, we analyzed white matter maturation adjusted for duration of TPN days. Remained significance, after adjustment for duration of TPN days, implies NEC is an independent risk factor for NDI; however, malnutrition may simultaneously increase the risk of worsening neurodevelopmental outcome.

This study had several limitations. One major limitation is the small sample size in the NEC group, which may lead to subject heterogeneity and skewed interpretation of the data. Greater statistical power could be gained by more balanced sample size or multicenter imaging data. Second, due to poor quality images with motion artifacts, a substantial amount of data was discarded during image processing; thus, caution must be exercised in the interpretation of results. However, in this study, all scans were performed during natural sleep, and all infants were carefully monitored with pulse oximetry and supervised by a skilled physician without sedation to ensure safety during the MRI scanning procedure. Compared with images of adults, there are many methodological challenges in obtaining neonatal MRI images. Finally, important questions remain regarding the contribution of genetic and postnatal environmental factors to the link between DTI findings and NDI. Although we corrected for important confounders such as GA and TPN days of nutrition, variables reflecting inflammatory status, including culture-proven sepsis and BPD, might have affected NDI.

## Conclusions

Preterm infants with evident NEC had delayed white matter maturation in the TEA. The delayed white matter maturation of sCC in the developing preterm brain was related to lower developmental scores at 18 months of CA, implying the relevance of sCC integrity as a biomarker of later NDI. Our results suggest that preterm infants with no evident brain injury may exhibit motor and language disorders with compromised structural connectivity in DTI. These findings would be strengthened and elaborated by large-population and long-term follow-up studies on how preterm infants overcome delayed maturation and altered white matter connectivity throughout life.

## Data Availability

The datasets used and analyzed during the current study are available from the corresponding author on reasonable request.

## Abbreviations

NEC: Necrotizing enterocolitis
NICU: Neonatal intensive care unit
NDI: Neurodevelopmental impairment
DTI: Diffusion tensor imaging
CA: Corrected age
TEA: Term-equivalent age
IUGR: Intrauterine growth retardation
IVH: Intraventricular hemorrhage
PVL: Periventricular leukomalacia
SIP: Spontaneous intestinal perforation
MRI: Magnetic resonance imaging
RDS: Respiratory distress syndrome
PDA: Patent ductus arteriosus
BPD: Bronchopulmonary dysplasia
ROP: Retinopathy of prematurity
TPN: Total parenteral nutrition
PMA: Postmenstrual age
BSID-III: The Bayley Scales of Infant and Toddler Development-III
BET: Brain extraction tool
FDT: FMRIB’s Diffusion Toolbox
FA: Fractional anisotropy
MD: Mean diffusivity
gCC: Genu of the corpus callosum
sCC: Splenium of the corpus callosum
ltCST: Left corticospinal tract
rtCST: Right corticospinal tract
ltILF: Left inferior longitudinal fasciculus
rtILF: Right inferior longitudinal fasciculus
